# Long‐acting and extended‐release implant and nanoformulations with a synergistic antiretroviral two‐drug combination controls HIV‐1 infection in a humanized mouse model

**DOI:** 10.1002/btm2.10237

**Published:** 2021-06-26

**Authors:** Jagadish Beloor, Shalley N. Kudalkar, Gina Buzzelli, Fan Yang, Hanna K. Mandl, Jyothi K. Rajashekar, Krasimir A. Spasov, William L. Jorgensen, W. Mark Saltzman, Karen S. Anderson, Priti Kumar

**Affiliations:** ^1^ Department of Internal Medicine, Section of Infectious Diseases Yale University School of Medicine New Haven Connecticut USA; ^2^ Department of Pharmacology Yale University School of Medicine New Haven Connecticut USA; ^3^ Department of Molecular Biophysics and Biochemistry Yale University School of Medicine New Haven Connecticut USA; ^4^ Department of Biomedical Engineering Yale University New Haven Connecticut USA; ^5^ Department of Chemistry Yale University New Haven Connecticut USA

**Keywords:** Compound I, drug synergy, EFdA, HIV, humanized mice, implants, long‐acting formulations, nanoformulations, pharmacokinetics

## Abstract

The HIV pandemic has affected over 38 million people worldwide with close to 26 million currently accessing antiretroviral therapy (ART). A major challenge in the long‐term treatment of HIV‐1 infection is nonadherence to ART. Long‐acting antiretroviral (LA‐ARV) formulations, that reduce dosing frequency to less than once a day, are an urgent need that could tackle the adherence issue. Here, we have developed two LA‐ART interventions, one an injectable nanoformulation, and the other, a removable implant, for the delivery of a synergistic two‐drug ARV combination comprising a pre‐clinical nonnucleoside reverse transcriptase inhibitor (NNRTI), Compound I, and the nucleoside reverse transcriptase inhibitor (NRTI), 4′‐ethynyl‐2‐fluoro‐2′‐deoxyadenosine. The nanoformulation is poly(lactide‐*co*‐glycolide)‐based and the implant is a copolymer of ω‐pentadecalactone and *p‐*dioxanone, poly(PDL‐*co*‐DO), a novel class of biocompatible, biodegradable materials. Both the interventions, packaged independently with each ARV, released sustained levels of the drugs, maintaining plasma therapeutic indices for over a month, and suppressed viremia in HIV‐1‐infected humanized mice for up to 42 days with maintenance of CD4^+^ T cells. These data suggest promise in the use of these new drugs as LA‐ART formulations in subdermal implant and injectable mode.

## 1 INTRODUCTION

Antiretroviral therapy (ART) has revolutionized the treatment of HIV‐AIDS and the disease is no longer a death sentence. However, as ART does not cure, treatment must be life‐long. Nonadherence to daily oral ART medication remains a significant barrier to achieve long‐term suppression of HIV replication and prevention of the emergence of drug‐resistant virus. Numerous studies show direct correlation between ART adherence and reduction in viral loads and elevation in CD4 T cell counts.^1^, ^2^, ^3^ Side‐effects and psychological reactions to taking ART also leads to nonadherence in HIV‐positive subjects, particularly younger individuals,^4^, ^5^ who account for more than half of all new HIV infections.^6^ With the exception of the recently approved Cabenuva, an injectable extended‐release nanoformulation of the integrase strand transfer inhibitor (INSTI), cabotegravir, and the nonnucleoside reverse transcriptase inhibitor (NNRTI) rilpivirine, there are no other approved ART formulations with a dosing frequency of less than once daily. Thus, new drugs with improved pharmacological properties, and a long‐acting (LA) antiretroviral (ARV) drug delivery system that can reduce the dosing frequency to weekly, monthly, or even longer periods of time could represent a significant advance in HIV treatment, especially in high‐risk populations.

**FIGURE 1:**
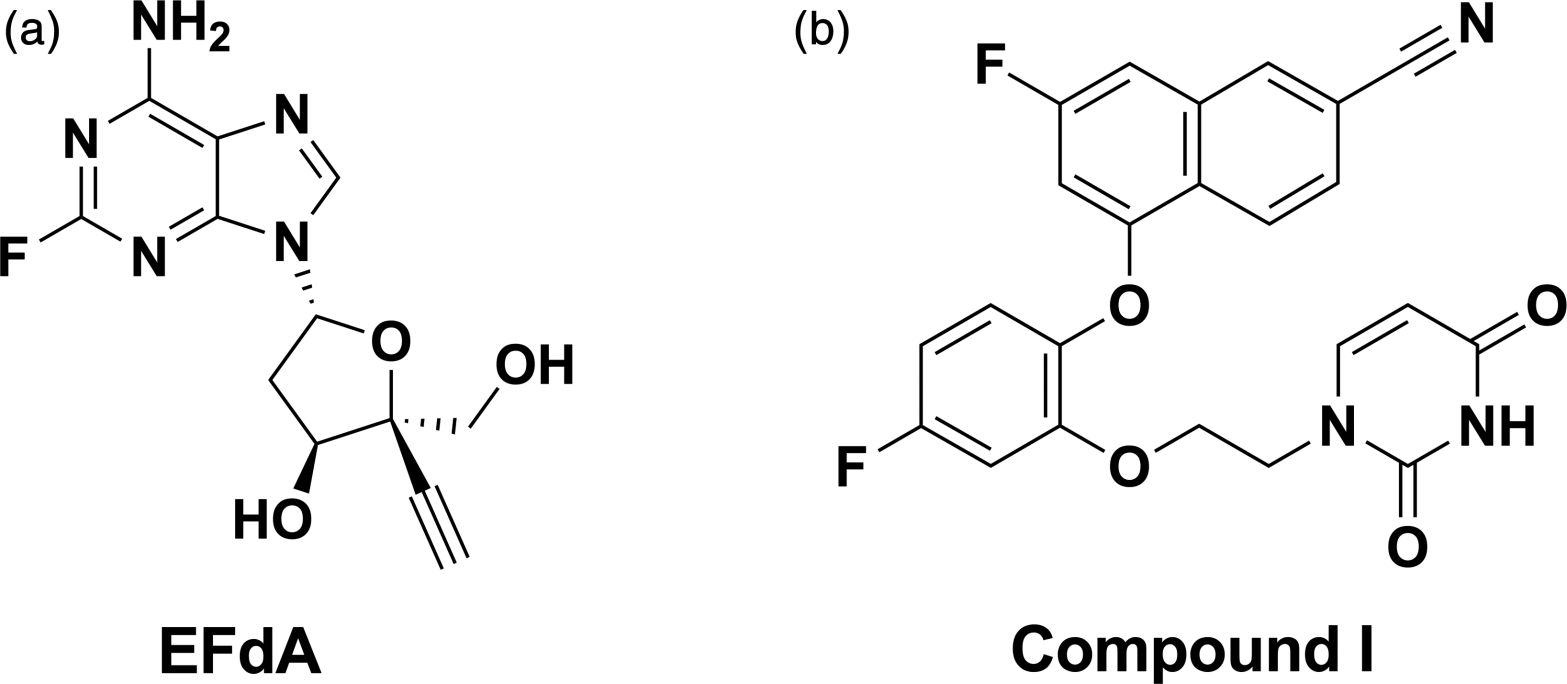
Chemical structures of (a) 4′‐ethynyl‐2‐fluoro‐2′‐deoxyadenosine (EFdA) and (b) Compound I

The majority of approved and investigational ARVs with sufficient antiviral potency are difficult to adapt for LA formulations due to suboptimal physicochemical properties as well as short plasma half‐lives, necessitating high drug loading (DL). Cabenuva, approved by FDA in January 2021, comprises once‐monthly intramuscular injections of rilpivirine and cabotegravir each, in a large (3 ml) volume, to achieve the desired pharmacokinetic profile.^7^, ^8^ An additional critical drawback of injectable LA nanoformulations is that they cannot be removed from the body in case of a medical emergency and can magnify adverse effects because of their intrinsic property of extending the half‐life of plasma drug concentrations.^9^ Some of these limitations may be addressed by removable biodegradable implants, which can also provide extended drug release over many months.^10^ Currently, 4′‐ethynyl‐2‐fluoro‐2′‐deoxyadenosine (EFdA; Figure 1(a)) (also called MK‐8591 and Islatravir), an investigational nucleoside reverse transcriptase inhibitor (NRTI) and dolutegravir (DTG), an INSTI, have been tested after formulating in removable, LA implants.^10^, ^11^ For EFdA implants, pharmacokinetic studies in rodents showed sustained therapeutic levels of the drug for up to 6 months although antiviral efficacy was not reported.^11^ The DTG implants delivered up to 9 months and showed some viral suppression and protection after vaginal challenge in animal models; however, drug resistance and viral breakthrough were noted as early as 19 days post‐therapy.^10^ These studies indicate promising results for removable HIV drug implants but reveal the limitations of monotherapy and the critical need for a multidrug combination, as in oral ART regimens.

The long‐term experience and success with three‐drug HIV therapeutic regimens, all of which contain two NRTIs in combination with an NNRTI, INSTI or a protease inhibitor, is well documented.^12^ While the only two approved two‐drug regimens do not include NRTIs,^12^ a new two‐drug NRTI/NNRTI regimen is currently being evaluated by Merck in Phase III clinical trials (ClinicalTrials.gov Identifier: NCT04233879). Many of these agents have shown synergistic antiviral potency in cell culture.^13^ Our previous studies showed potent synergy between a preclinical candidate NNRTI, Compound I, which is a catechol diether and the NRTI, EFdA (Figure 1(a,b)).^14^, ^15^, ^16^, ^17^ Development of Compound I was guided by mechanistic studies and computational design leading to enhanced pharmacological properties, drug resistance profiles, and a wide margin of safety relative to the current FDA approved NNRTIs such as efavirenz and rilpivirine. Compound I is active in the nanomolar range against wild‐type HIV‐1 strains and common drug resistant variants including Y181C and K103N, and demonstrates synergistic antiviral activity with existing HIV‐1 drugs and clinical candidates, notably EFdA.^13^, ^14^, ^15^, ^16^, ^17^ Compound I also demonstrates favorable pharmacokinetics and absorption, distribution, metabolism, excretion, toxicity (ADME‐Tox) profile as well as antiviral efficacy in a hu‐mouse model for HIV‐1.^13^, ^18^ Notably, Compound I showed no inhibition of the HERG ion channel which might prolong the Q‐T interval leading to cardiotoxicity which has limited the dosing of the NNRTI rilpivirine.^18^ Furthermore, a poly(lactide‐*co*‐glycolide) (PLGA) nanoformulation of Compound I enabled sustained maintenance of plasma drug concentrations and antiviral efficacy for 3–5 weeks after a single dose.^13^

In this report, we extend on these observations using the PLGA‐based nanoformulation to deliver the synergistic two‐drug combination of Compound 1 and EFdA for an extended release profile in hu‐mice. Compound I and EFdA were chosen as the NNRTI and NRTI combination, based upon their optimal pharmacological ADME‐Tox properties, potent and synergistic antiviral efficacy in suppressing viral replication. EFdA has been one of the most potent NRTIs evaluated and the clinical trials conducted by Merck have shown very promising results.^19^, ^20^

We further advanced the formulation to implants with copolymers of ω‐pentadecalactone and *p‐*dioxanone, poly(PDL‐*co*‐DO), a novel class of biocompatible, biodegradable materials that maintain structural integrity during degradation and also allow for ready removal should issues with toxicity arise. Poly(PDL‐*co*‐DO) has physical properties particularly well suited for providing long‐term sustained release not achievable with other polymers that degrade via hydrolysis such as PLGA and other polyesters.^21^, ^22^, ^23^ Poly(PDL‐*co*‐DO) is semicrystalline over the entire range of copolymer compositions; varying the copolymer composition alters its degradation rate, drug release rate. Importantly, degradation is slow enough to allow production of a degradable device that remains mechanically strong for at least 12 months. Previously, we showed that films of poly(PDL‐*co*‐DO) are well‐tolerated after subcutaneous implantation and that poly(PDL‐*co*‐DO) can be formulated into particles that slowly release doxorubicin or siRNA.^23^ We have also demonstrated that poly(PDL‐*co*‐DO) can be engineered into a degradable contraceptive implant that provides consistent release of levonorgestrel for periods of over 2 years.^24^

The current report describes the efficacy of Compound I/EFdA combination, independently formulated as LA PLGA‐based nanoformulations as well as poly(PDL‐*co*‐DO) implants in terms of pharmacokinetics and antiviral efficacy in a hu‐mouse model of HIV infection.

## 2 MATERIALS AND METHODS

### 2.1 Materials

Synthesis of Compound I has been reported.^14^, ^16^ EFdA was custom synthesized by WUXI, Shanghai, China. Poly(d,l lactic‐*co*‐glycolic acid) acid‐terminated, 75:25 with MW 4000–15,000 was acquired from Evonik for fabrication of EFdA‐loaded nanoparticles (NPs). Poly(d,l lactic‐*co*‐glycolic acid), 50:50 with inherent viscosity 0.55–0.75 dl/g was used for fabrication of Compound I‐loaded NPs. Poly(PDl‐*co*‐DO)was synthesized and analyzed as reported previously.^21^

### 2.2 Fabrication of Compound I and EFdA‐NPs

Compound I‐loaded PLGA NPs was formulated by a single emulsion‐solvent evaporation method.^13^, ^25^

Due to the hydrophilic properties of EFdA, EFdA‐loaded PLGA NPs were formulated using a water‐in‐oil‐in‐water (W‐O‐W) technique.^26^, ^27^ Then, 100 mg of PLGA was dissolved in 5 ml of DCM overnight (final −20 mg/ml). The inner aqueous phase of the W‐O‐W emulsion comprised 12.04 mg of EFdA dissolved in 2 ml of 1 mM HEPES buffer (pH 9.0). This was added dropwise under constant magnetic stirring to the organic phase of 5 ml PLGA solution and 2 ml of 1% Pluronic F‐127. The mixture was sonicated for 10 s using a probe sonicator. To form the W‐O‐W emulsion, the water‐in‐oil (WO) phase was added to 20 ml of the outer aqueous phase (1% PVA solution) and sonicated for 10 s. DCM was evaporated, NPs washed, and lyophilized as above.

### 2.3 Characterization of Compound I and EFdA NPs

DL was determined by dissolving a known mass of lyophilized NP in DMSO. The samples were filtered through an Acrodisc 25‐mm syringe filter with a 0.45‐μm HT Tuffryn membrane (Pall Life Sciences) followed by additional dilution in acetonitrile (ACN). The samples were analyzed using HPLC as described.^18^ The limit of detection (LOD) for Compound I and EFdA was 0.1 and 0.25 μg/ml, respectively.

NP size, PDI, and zeta (**ζ**) potential were measured by dynamic light scattering by resuspending 0.05 mg NP in 1 ml deionized water using a Zetasizer Nano ZS90 (Malvern Instruments). SEM images were obtained on a Hitachi SU7000 scanning electron microscope. To measure surface charge (**ζ**), NPs were diluted in deionized water at a concentration of 0.5 mg/ml; 750 μl of solution was loaded into a disposable capillary cell (Malvern Instruments), and the charge measured using a Malvern Nano‐ZS.

### 2.4 Formulation of Compound I and EFdA containing LA subdermal implants

Poly(PDL‐*co*‐DO) with 40% *p‐*dioxanone (DO) content (mol%) and a molecular weight of 51,178 Da was used. For Compound I implants, poly(PDL‐*co*‐DO) (180 mg) and Compound I (120 mg) were dissolved in 10 ml of a 50:50 DCM:chloroform mixture. For EFdA implants, poly(PDL‐*co*‐DO) (180 mg) and EFdA (120 mg) were dissolved in 10 ml of DCM in a glass vial. A rotary evaporator was used to completely evaporate the DCM and chloroform over ~20 min. The resulting drug/polymer film was lyophilized to remove excess water. The film (~120 mg) was then loaded into a Teflon mold and baked for 1 h at 90°C under argon protection and atmospheric pressure to form implants. Immediately after baking, the implants were compressed overnight using a stainless steel plunger. The resulting implants were 2 cm in length and approximately 100 mg in weight. The implants were then cut to 1 cm in length and weighed.

### 2.5 Characterization of drug‐loaded implants

For the Compound I implant, the sample was dissolved in 1 ml of DCM, and DCM was evaporated under a steady stream of nitrogen. For the EFdA implant, the sample was dissolved in 1 ml of chloroform, and 1 ml water was added to it. The mixture was vortexed and let it sit to extract the drug into the water phase. Centrifugation was carried out to separate layer of chloroform and water. The upper water layer containing the sample was removed and lyophilized to dry out the water. The dried pellets were dissolved in ACN for HPLC analysis. The LODs have been detailed above. Reverse‐phase HPLC was used to measure DL (%), defined as the measured mass of Compound I per mass of PLGA NP/implants, and EE (%), which is defined as the ratio of the compounds loaded to the total drugs used for fabricating the NPs/implants as described earlier.^18^

Compound I and EFdA implants were also evaluated under an ultra‐high‐resolution Hitachi scanning electron microscopy (SU7000). Implants were flash frozen in liquid nitrogen. Implants were then broken in half using tweezers and cross‐sectioned using a razor blade to about 1 mm thickness. Samples were placed on a stub using carbon tape, with razor blade edge facing down. Samples were coated with platinum to a thickness of 5 nm using a high resolution sputter coater (Cressington, 208HR) with rotary planetary tilt stage and thickness controller MTM‐20.

### 2.6 Generation of hu‐mice

NOD.Cg‐Prkdcscid Il2rgtm1Wjl/SzJ (NSG) mice were purchased from the Jackson Laboratory (Bar Harbor, ME). All experiments were performed according to protocols approved by the Institutional Review Board and the Institutional Animal Care and Use Committee of Yale University. The animal model used in these studies is the NSG‐Hu‐PBL as described.^28^

### 2.7 Administration of NPs

Hu‐mice were injected intraperitoneally (i.p.) with Compound I‐NP (190 mg/kg) and EFdA (10 mg/kg) suspended in sterile saline solution. The i.p. route was chosen based on our previous studies with Compound I as a free drug solubilized in 10% DMSO/detergent.^18^ The dosing was based on in vivo efficacy with free drug and calculated taking into account the amount of each ARV loaded in the NP formulation.

### 2.8 Implantation procedure

Hu‐mice were anesthetized by i.p. injection of ketamine (80 mg/kg) and xylazine (12 mg/kg) in PBS (10 ml/kg) based on individual mouse body weight. After anesthetizing, approximately 1–2 cm square of the dorsal skin was shaved using an electric hair clipper, the area was cleaned with ethanol and disinfected with Betadine. Using a sterile disposable surgical blade, an incision of 4–5 mm was made through the skin. Gently, 2 cm × 3 cm subcutaneous pockets were created with forceps and implants loaded with Compound I and EFdA were co‐inserted at the same site. The opening was sutured using absorbable sutures followed by subcutaneous administration of 0.05 mg/kg buprenorphine. The animals were kept warm using the temperature‐controlled heating pads until they regained consciousness. Toxicity was evaluated by clinical observations, cage‐side observations (twice daily), and body weight (at least weekly).

### 2.9 In vivo pharmacokinetic studies for implants and NPs

Blood samples were collected at predetermined time points from the ocular venous plexus by retro‐orbital venipuncture and serum was used for subsequent HPLC analysis as detailed previously.^18^

### 2.10 HIV‐1 challenge experiments in hu‐mice

Hu‐PBL mice that were i.p. injected with NPs or surgically inserted with implants were i.p. challenged with 30,000 FFU of HIV‐1_JRCSF_. The kinetics of virus infection were monitored by weekly measurements of plasma viral loads (PVLs) and peripheral blood CD4 T cell in longitudinal bleeds as described previously.^13^

### 2.11 Analysis of ARV activity in serum from drug administered mice

Sera from mice were heat‐inactivated at 56°C, diluted at 1:20 or 1:100 in complete DMEM and added to 15,000 TZM‐bl cells seeded 24 h earlier per well in a 96‐well plate. After 2 h, cells were infected with HIV‐1_JRCSF_ (multiplicity of infection, 0.1). After 48 h, cells were lysed and luciferase activity measured using a luciferase assay kit (Promega) following manufacturer's protocol. Inhibition percentages in serum samples from implants and NP‐administered mice were calculated relative to the read‐out from the control uninfected Hu‐PBL mouse serum.

### 2.12 Statistical analysis

The data were plotted as a concentration‐time curve using PRISM 9.0 (GraphPad Software Inc., La Jolla, CA). EC_50_ was calculated using Graph pad prism where EC_50_ of each compound was used along with Hill slope of 1. The predicted area under the curve (AUC) for drug concentration in blood serum against time (AUC predicted) was calculated based on the linear trapezoid method.^29^ AUC was also calculated for PVLs and CD4+ T‐cells in infection experiments. Wilcoxon signed rank test was performed to test any statistical significance between controls and drug treated mice.

## 3 RESULTS

### 3.1 Development of LA nanoformulations

Our previous studies indicated that a single dose of LA‐NPs released sustained levels of Compound I for ∼3 weeks and effectively controlled viral loads in HIV‐1_JRCSF_‐infected hu‐mice.^13^ Given the intended use of Compound I in combination therapy with EFdA, and the distinct physiochemical properties of the two drugs (Compound I is hydrophobic while EFdA is hydrophilic), the two drugs were independently formulated as PLGA NPs.

**FIGURE 2:**
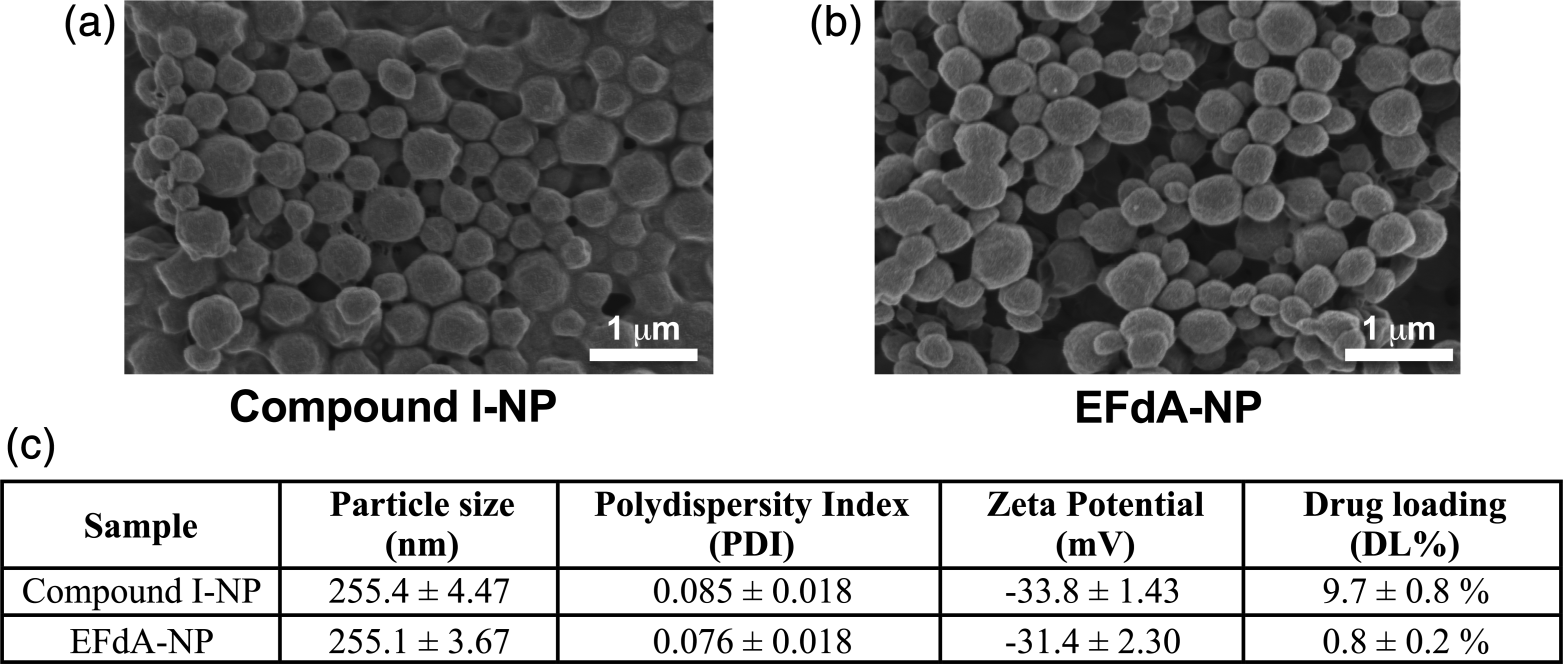
Characterization of long‐acting (LA)‐nanoparticle (NP) formulations. (a) Physical characteristics. Data shown are mean ± SD of four independent sample measurements. (b,c) Scanning electron microscopy images of Compound I‐NP (b) and 4′‐ethynyl‐2‐fluoro‐2′‐deoxyadenosine (EFdA)‐NP (c). The scale bar as depicted is 1 μm

The physical characteristics of Compound I‐NP and EFdA‐NP are depicted in Figure 2. The nanoformulations exhibited an average diameter of 255 ± 4.5 nm for Compound I‐NP and 255 ± 3.7 nm for EFdA‐NP, with a polydispersity index of <0.1 and an average negative zeta potential of −33.8 and −31.4 for Compound I‐NP and EFdA‐NP, respectively. Representative SEM images illustrated that Compound I‐NP (Figure 2(a)) and EFdA‐NP (Figure 2(b)) exhibited fairly uniform spherical shapes. We undertook to achieve a DL of 10 wt% for each drug in the NP formulation; we attained 9.7 ± 0.8 wt% for Compound I; however, due to the hydrophilicity of EFdA, the resultant DL achieved was 0.8 ± 0.2 wt% (Figure 2(c)).

### 3.2 Pharmacokinetics of drug release and antiviral efficacy of Compound I and EFdA nanoformulations in Hu‐mice

**FIGURE 3:**
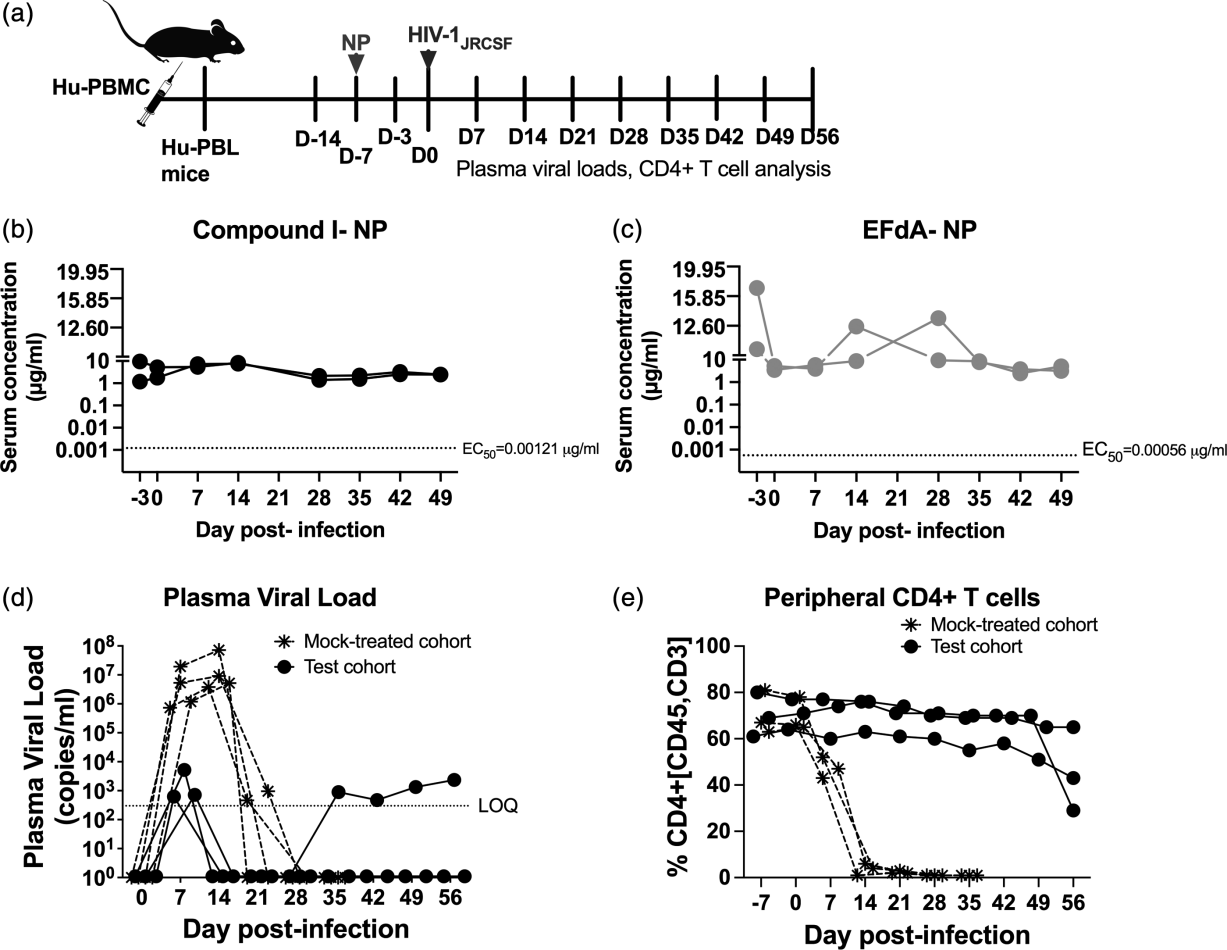
(a) Experimental timeline for studies with nanoformulations in HIV‐1_JRCSF_‐infected Hu‐PBL mice. (b,c) Serum concentrations of (b) Compound I and (c) 4′‐ethynyl‐2‐fluoro‐2′‐deoxyadenosine (EFdA) in Hu‐PBL mice coadministered Compound I‐nanoparticle (NP) and EFdA‐NP. (d,e) Hu‐PBL mice were infected with HIV‐1_JRCSF_ 7 days postadministration of Compound I‐NP and EFdA‐NP; test cohort, (solid black circles) and control cohort (mock‐treated, asterisk). Mice were bled weekly for (d) plasma viral load (PVL) analyzed by qPCR and (e) CD4+ T cells analyzed by flow cytometry. Each line graphed represents one mouse. The dashed line intercept in (b) and (c) depicts cellular EC_50_ of Compound I and EFdA, respectively, and in (d) denotes limit of quantitation of HIV‐1 viral RNA

In vivo drug release from NPs were analyzed in Hu‐PBL mice, generated by reconstituting NSG mice with healthy human PBMC. LA‐NPs containing either EFdA or Compound 1 were coadministered i.p. as a mixture in Hu‐PBL mice 7 days prior to infection (Day −7 in experimental timeline, Figure 3(a)) with 30,000 infectious units of HIV‐1_JRCSF_ (D0). The experimental timeline was based on our previous efficacy study^13^ and other studies exploring efficacy of ARVs in humanized mice.^20^, ^30^, ^31^, ^32^, ^33^ NP were administered at a single dose of 190 mg/kg for Compound I and 10 mg/kg for EFdA, which had yielded sustained in vivo drug concentrations above EC_50_ (2.8 nM [1.21 ng/ml] for Compound I and 1.9 nM [0.557 ng/ml] for EFdA in cellular assays) in our recent study.^13^, ^20^ Serum levels of Compound I and EFdA prevailed above EC_50_ at 4 days post‐NP administration in two mice of the cohort that were tested and all mice were infected with HIV‐1_JRCSF_ on Day 7 (Day 0 in Figure 3(a)). As before, the NPs, sustained serum levels of Compound I and EFdA above EC_50_ for at least 49 days, at which time, the experiment was terminated in accordance with the experimental timeline admissible with Hu‐PBL mice (Figure 3(b,c)). Higher serum drug concentrations, averaging at 10.6 and 13.6 μg/ml for Compound I‐NP and EFdA‐NP, respectively, were observed 4 days postadministration, presumable as a declining aftermath of the transient burst in drug release that is expected to occur after NP administration.^13^, ^20^ A 15% increase in Compound I and a 21% increase in EFdA serum concentrations was observed at Day 21 post‐NP administration (14‐day postinfection). This level was maintained until Day 28 for EFdA followed by a twofold decline over the next 3 weeks. Compound I serum concentration decreased by sevenfold at Day 28 and was maintained at that level until the end of the study. The observed AUC_0‐last_ for Compound I‐NP and EFdA were 3874 ± 243.1 μg × h/ml and 6978 ± 518 μg × h/ml with a clearance (CL) of 0.8 ml/min/kg and 0.02 ml/min/kg, respectively (Table 1). As before, no toxicities were induced by the nanoformulations.^13^

**TABLE 1:** Pharmacokinetic parameters of Compound I‐NP and EFdA following i.p. injection of formulations in humanized mice

Pharmacokinetic parameters	Compound I‐NP	EFdA‐NP	Compound I‐implant	EFdA‐implant
Dose (mg/kg)	190	10	406 ± 105	757 ± 151
AUC_0‐last_ (μg h/ml)	3874 ± 243.1	6978 ± 518	10,058 ± 2074	9129 ± 1323
CL (ml/min/kg)	0.8	0.02	0.6	1.3

Abbreviations: AUC, area under the curve; CL, clearance; EFdA, 4′‐ethynyl‐2‐fluoro‐2′‐deoxyadenosine; i.p., intraperitoneally; NP, nanoparticle.

Antiviral efficacy was assessed by measuring the PVLs and peripheral CD4+ T‐cells postinfection on weekly basis by collecting blood samples retro‐orbitally. HIV‐1 RNA was detected in plasma of all three mice of each cohort 1 week after exposure with high levels in the control, mock‐treated group (median 3.24 × 10^6^ copies/ml, range 0.73–19.2 × 10^6^ copies/ml, Figure 3(d)). This was accompanied with a rapid loss in human CD4+ T cells, an indicator of pathogenic effects caused by the HIV‐1 infection (Figure 3(e)). PVLs in control mice declined to below the level of quantitation (LOQ, 150 copies of HIV‐1 RNA/ml plasma) by 3 weeks postinfection, concomitant with the complete loss of human CD4+ T cells that are required for viral replication and titers (Figure 3(d)). While productive infection did occur in the test cohort of mice, the PVLs were 3 log units lower (median 0.72 × 10^3^ copies/ml, range 0.71–5.19 × 10^3^ copies/ml, Figure 3(d)). PVLs fell to below LOQ and were maintained at these levels for 3–4 weeks (Figure 3(d)). PVLs rebounded in one mouse at Day 35 and translated to a drop in CD4+ T cells by Day 49 (Figure 3(d)). On an average, a reduction of four log_10_ was observed in the test cohort compared to the highest PVLs attained in the control group at D14 indicating continued HIV‐1 inhibition in these mice. In both mice, >85% of CD4+ T cells were protected throughout the study relative to D0 (Figure 3(e)). PVL AUC was significantly smaller in the test cohort compared to the control group (*p* = .05; Wilcoxon signed rank test).

### 3.3 Development of LA implants

**FIGURE 4:**
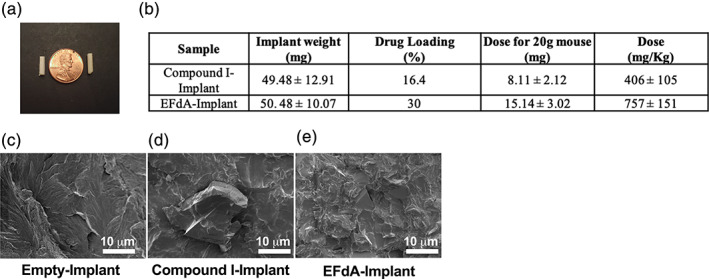
Characterization of long‐acting (LA)‐implant formulations. (a) Implant image depicting the size relative to a penny. (b) Physical characteristics of LA‐implants formulated with Compound I or 4′‐ethynyl‐2‐fluoro‐2′‐deoxyadenosine (EFdA). Data shown are mean ± SD of four independent sample measurements. (c,d) Representative high‐resolution SEM images showing a single cross section through the diameter of (b) empty (c) Compound I‐loaded, and (d) EFdA‐loaded implants. The scale bar as depicted is 1 μm

The poly(PDL‐*co*‐DO) implants were again formulated independently with the EFdA and Compound I because of their distinct physiochemical properties. Both implants were ~1 cm long and off‐white in color (Figure 4(a)). Figure 4(b) depicts the physical characteristics of each implant. EFdA loading efficacies in the implant were significantly better than in the NP formulations and the average calculated percent loading per implant was 16.4% for Compound I and 30% for EFdA. The implants were characterized using SEM to assess their morphology and uniformity (Figure 4(c–e)). Compared to the empty polymer implants, which displayed a continuous gray region by SEM, the Compound I‐ and EFdA‐loaded implants had regions that appeared brighter, presumably due to the presence of drug or drug crystals.

### 3.4 Pharmacokinetics of drug release and antiviral efficacy of Compound I‐ and EFdA‐loaded implants in Hu‐mice

**FIGURE 5:**
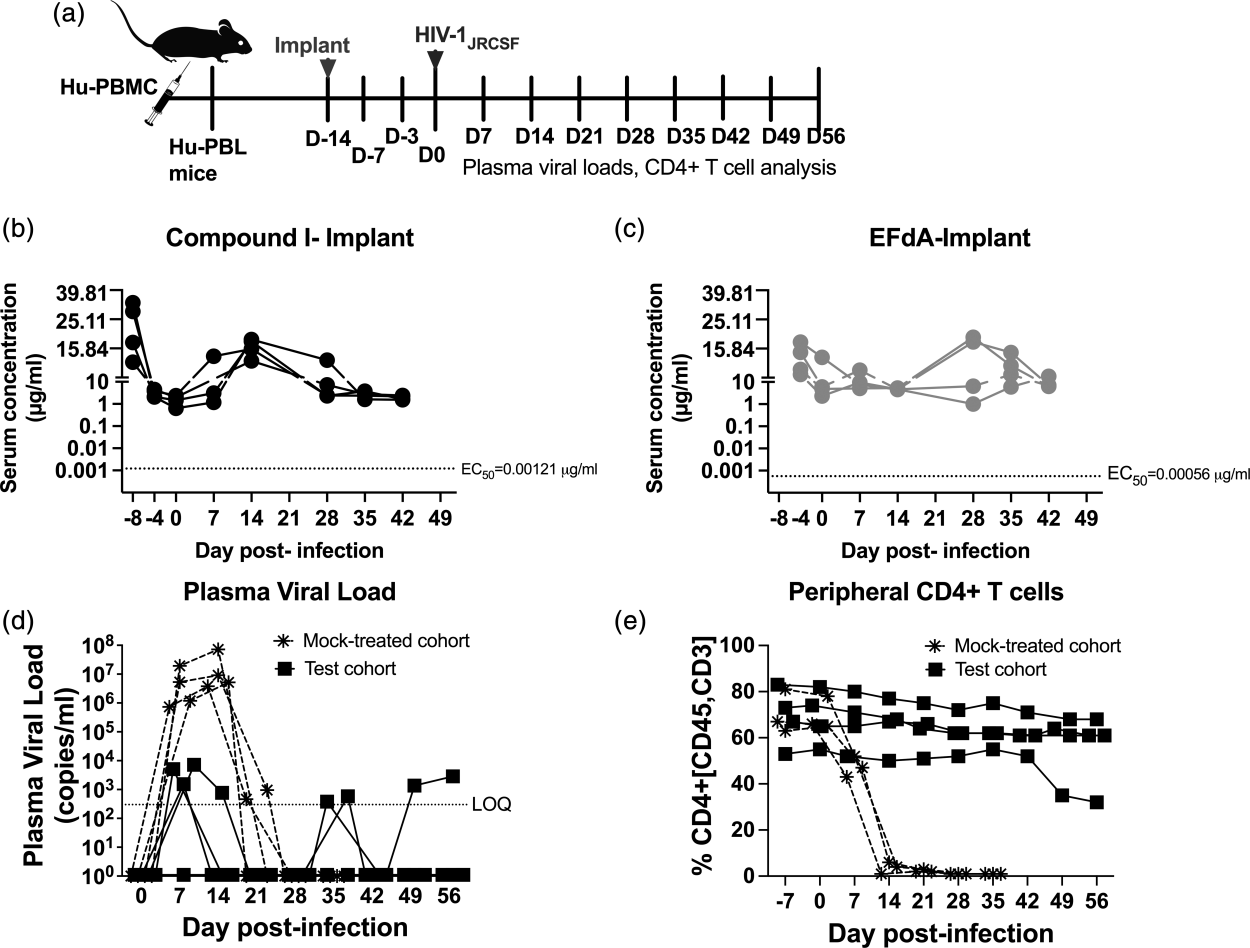
(a) Experimental timeline for studies with implants in HIV‐1_JRCSF_‐infected Hu‐PBL mice. (b,c) Serum concentrations of (b) Compound I and (c) 4′‐ethynyl‐2‐fluoro‐2′‐deoxyadenosine (EFdA) in Hu‐PBL mice with Compound I‐ and EFdA‐loaded implants. (d,e) Hu‐PBL mice were infected with HIV‐1_JRCSF_ 14 days postimplant; test cohort, (solid black squares) and control cohort (mock‐treated, asterisks). Mice were bled weekly for (d) plasma viral load (PVL) analyzed by qPCR and (e) CD4+ T cells analyzed by flow cytometry. Each line graphed represents one mouse. The dashed line intercept in (b) and (c) depicts cellular EC_50_ of Compound I and EFdA, respectively, and in (d) denotes limit of quantitation of HIV‐1 viral RNA

Implants loaded with Compound I and EFdA were surgically inserted at 14 days prior to HIV‐1_JRCSF_ challenge (D‐14, Figure 5(a)). The profile of serum drug concentrations in hu‐mice harboring the two drug‐loaded implants are shown in Figure 5(b,c). In both cases, there was evidence of an initial transient burst in drug release. For mice with Compound I‐implants, serum concentrations ranged between 12.7 at 32.5 μg/ml at Day 7 postimplantation (Figure 5(b)). In mice with EFdA‐implants, the range spanned 10.5–17.5 μg/ml at Day 10 postimplantation (Figure 5(c)). While serum drug levels due to release from the implants fluctuated through the period of observation (for instance, median serum concentrations for Compound I on Days 14 and 28 postimplant ranged from 1.7 to 16.7 μg/ml and for EFdA from 4.6 to 12.0 μg/ml on Days 28 and 42 postimplant), they were always well above the EC_50_ necessary to maintain viral suppression (Figure 5(b,c)). For instance, levels for Compound I on Day 14 and EFdA on Day 28 were at least 1400‐ and 8363‐fold higher than the cellular EC_50_ values. Overall, Compound I and EFdA implants sustained serum drug levels at greater than IC_90_ (17.1 and 25.2 nM, respectively) starting at Days 7–10 days after implant and maintained these levels till at least 56 days when the study was terminated. The calculated AUC_0‐last_ for Compound I was 10,058 ± 2074 μg × h/ml with 95% confidence interval of 5993–14,124 μg × h/ml and for EFdA 9129 ± 1323 μg × h/ml with 95% confidence interval of 6535–11,723 μg × h/ml (Table 1).

Overall, there were no significant abnormalities due to the implant, and the majority of observations noted were considered to be incidental, procedure related, or common findings for mice undergoing a surgical procedure. The incision sites healed normally within a few days after surgery. There was no evidence of inflammation, toxicity, or poor tolerability at the implantation sites throughout the duration of the study.

On Day 14 postimplant, the mice were infected with HIV‐1_JRCSF_. HIV‐RNA was detected in plasma of all exposed mice at the first sampling, a week after infection (Figure 5(d)). The control cohort implanted displayed high levels of plasma viremia (median 3.24 × 10^6^ copies/ml, range 0.73–19.2 × 10^6^ copies/ml). In contrast, PVLs in the implant group were 3 log units lower (median 3.31 × 10^3^ copies/ml, range 0–7.18 × 10^3^ copies/ml). Control mice maintained high concentrations of plasma HIV‐1 RNA for up to 2 weeks after which, PVLs declined, concomitant with the complete loss of human CD4+ T cells (Figure 5(d)). In contrast, viral loads fell to below LOQ, (150 copies of HIV‐1 RNA/ml plasma) in 3 out of 4 mice in the test cohort by Day 14 and the last mouse by Day 21. All mice with drug implants continued to maintain PVLs below LOQ until 4 weeks postinfection. On Day 35, two of the four mice in the implant group showed the transient presence of PVLs above the LOQ; however, these blips fell below LOQ for the remainder of the study. Viral rebound was observed in one other mouse toward the end of the study on Day 42 postinfection. PVL AUC was significantly smaller in the test cohort compared to the mock‐treated control (*p* < .05; Wilcoxon signed rank test).

The observations with PVLs were supported by the virus‐induced effects on peripheral CD4+ T cells (Figure 5(e)). To begin with, the average levels of CD4^+^ T‐cells on D0 prior to HIV infection in both groups were similar and ~70 ± 13%. Upon infection, mice in the control cohort, displayed a significant decline in CD4+ T cell percentages and by Day 14, lost >97% of these cells in all mice in this group. On the other hand, for mice in the test cohort, >85% of CD4+ T cells were protected throughout the study relative to Day 0. At the last time point, for implant (D56 pi), the only mouse with PVLs showed a significant decline in CD4+ T cells (~32%).

### 3.5 Inhibition of HIV‐1 replication ex vivo

**FIGURE 6:**
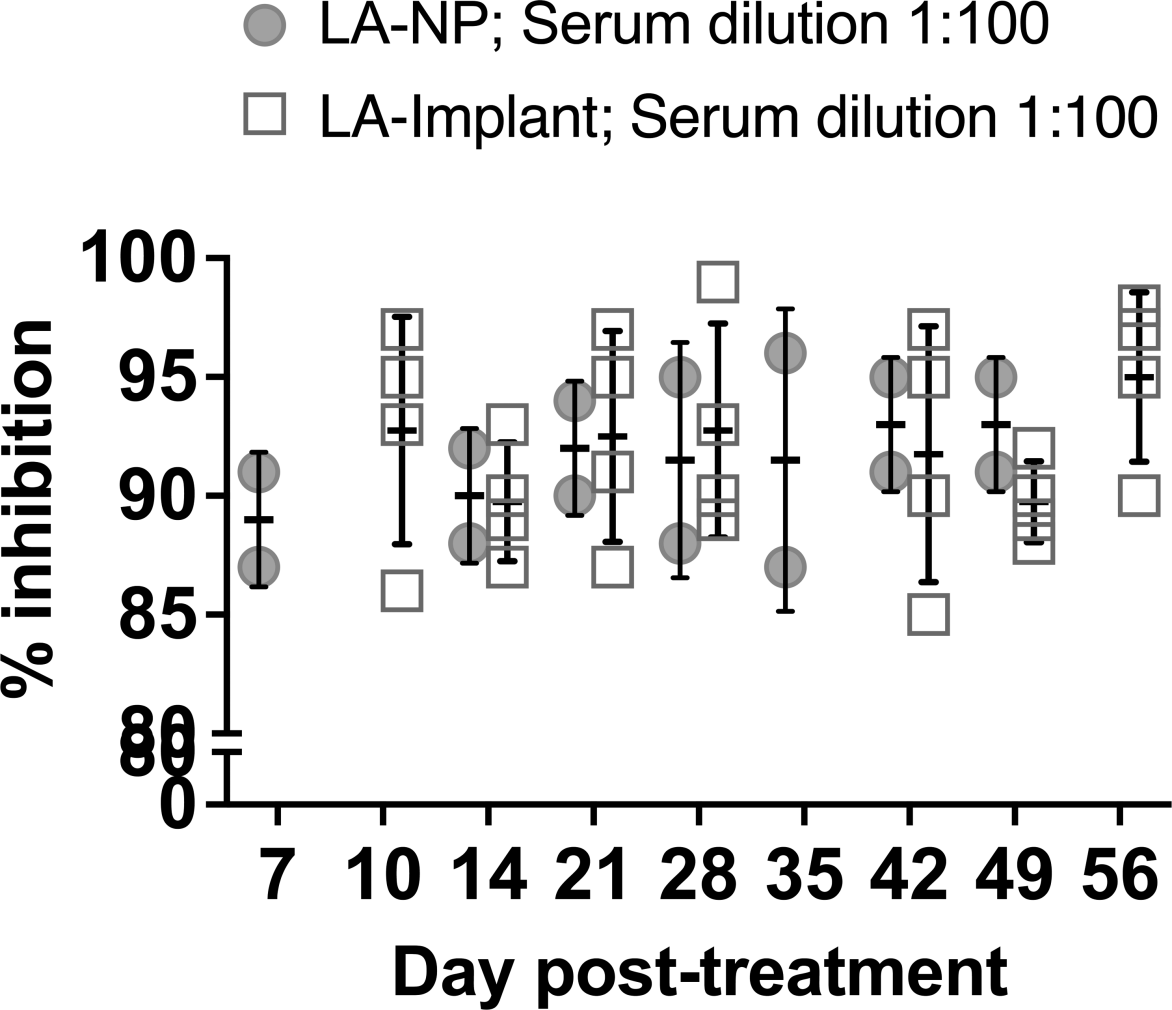
Inhibition of in vitro HIV‐1 infection with serum from Hu‐PBL mice undergoing Compound I/4′‐ethynyl‐2‐fluoro‐2′‐deoxyadenosine (EFdA) combination therapy supplied through Implants or nanoparticles (NPs). Each data point represents serum from a single mouse

Having demonstrated sustained concentrations of Compound I and EFdA in serum of hu‐mice, we evaluated antiviral activity ex vivo. Sera from the implanted mice demonstrated strong antiviral activity (Figure 6). Specifically, a 100‐fold dilution of every serum sample collected at each timepoint through the experimental timeline (Figures 3(a) and 5(a)) was able to block infection of TZM‐bl cells with HIV‐1_JRCSF_ by ~90% in vitro (median 91.75%, range 89.75–95.0%, *n* = 4, Figure 5). Importantly, the activity was comparable to a 1:20‐fold dilution of the serum (data not shown). A 100‐fold dilution of serum collected throughout the time period of testing from LA‐NP‐treated mice also blocked HIV‐1 infection of TZMBL cells by ~90% (median 91.50%, range 89.00–93.0%, *n* = 2, Figure 6).

## 4 DISCUSSION

Patient adherence to lifelong HIV therapeutic regimens is limited by the necessity of daily dosing of medications.^34^ Therefore, a number of recent research efforts have focused on long‐acting, extended release formulations summarized in a recent review.^9^ Cabenuva has currently been approved by the FDA.^8^ Further advances have been reported in preclinical studies with AIDS drugs formulated as LA, extended release implants.^9^ These include an in situ generated implant with DTG as a monotherapy for HIV treatment and prevention.^10^ A study in humanized mice with this implant showed sustained drug levels over almost 2 months and a one to two log drop in viral load; however, drug‐resistant HIV variants emerged after 19 days. In addition, several bio‐erodible and nonerodible implants containing EFdA as a single agent showed extended drug levels for over 6 months in rats and nonhuman primates although no efficacy studies were reported.^11^ In the current study, we describe the pharmacokinetics and antiviral efficacy of a synergistic combination of an NRTI and NNRTI formulated as LA formulations. The NRTI EFdA was selected due to its exceptional potency and lack of toxicity including mitochondrial toxicity observed with other NRTIs and promising Phase III clinical trials. The NNRTI, Compound I, is a computationally designed preclinical candidate chosen because of its excellent antiviral efficacy as a LA NP formulation, ADME‐Tox and drug resistance profiles as well as synergistic behavior with EFdA as a two‐drug combination in cell culture. The poly(PDL‐*co*‐DO) implant utilized for these studies has several desirable features for LA, sustained drug delivery. These attributes include: (1) biocompatibility and biodegradability while maintaining structural integrity in the event removal is required due to toxicity and (2) extended long‐term drug release potentially lasting for several years.

Both formulations, the LA‐NP and LA‐implant, showed sustained plasma concentrations for both drugs till the termination of study. Compound I and EFdA in sera were 2.0 μg/ml (4 μM) and 4 μg/ml (13 μM), respectively, at Day 49 in hu‐mice with nanoformulations, and ~2 μg/ml (6.8 μM) and 7.5 μg/ml (25 μM), respectively, at Day 56 in hu‐mice with implants. These were approximately 100‐ and 1100‐fold above the EC_90_ in HIV cell culture (25.2 nM [10 ng/ml] and 17.1 nM [5 ng/ml], respectively). The plasma concentration of EFdA was sustained above EC_50_ despite the 1% loading implants. Furthermore, analysis of PK data revealed that approximately twofold higher dosing achieved with the Compound I‐implant resulted in 2.5‐fold higher AUC_0‐last_ than the Compound I‐NP. The CL was unchanged for both the Compound I formulations. The higher volume of distribution accompanied with a low CL rate suggests slower metabolism of Compound I, thereby enabling sustained levels for viral control as shown in our previous studies with free Compound I.^18^ The AUC_0‐last_ for EFdA‐implant was only 1.3‐fold higher compared to EFdA‐NP despite a 75‐fold higher dose of EFdA loaded in the implant suggesting that the increased dose did not increase the volume of distribution of EFdA.

Nevertheless, the disparate PK parameters observed for LA‐NP and LA‐implant did not affect the efficacy of these two compounds. Both formulations displayed potent antiviral efficacy in hu‐mice, as evidenced by the drop in PVLs to below LOQ in treated mice during the period of observation and the ability of sera from all mice to completely inhibit HIV infection ex vivo. This was also supported by protection of viral‐induced depletion of peripheral CD4^+^ T cells in the test cohorts. However, it appears that productive infection occurred in all mice as evidenced by the detection of PVLs a week after infection and viral rebound in one mouse of both treated cohorts despite initiating treatment prior to virus challenge. Published studies exploring LA ARV formulations (in contrast to free‐formulated ARV) have also demonstrated prophylactic efficacy only when humanized mice are infected with HIV‐1 a week or more after initiating treatment with LA formulations.^31^, ^32^, ^33^ This is despite plasma drug levels reaching EC50 well within a week, whether this is because it takes longer to achieve stable intracellular and tissue EC50 drug concentrations needs to be investigated. It also needs to be investigated if the rebounding virus was due to viral escape, and the small serum sample volumes did not permit these studies.

## 5 CONCLUSION

The current study using poly(PDL‐*co*‐DO) implants or PLGA nanoformulations to deliver a two‐drug therapeutic regimen demonstrates the value of two synergistic antiviral compounds to completely suppress viral loads and protect CD4+ T cells. This approach significantly extends previous findings with a long‐acting implant containing DTG as monotherapy where the onset of drug resistance was found as early as Day 19 postinfection.^10^ Future considerations will include the use of hu‐mouse models that will permit evaluation of antiviral efficacy over even longer time periods.

## AUTHOR CONTRIBUTIONS

**Jagadish Beloor**: Conceptualization; data curation; formal analysis; investigation; methodology; validation; writing‐original draft; writing‐review and editing. **Shalley N. Kudalkar**: Conceptualization; data curation; formal analysis; investigation; methodology; validation; writing‐original draft; writing‐review and editing. **Gina Buzzelli**: Data curation; methodology; visualization; writing‐original draft; writing‐review and editing. **Fan Yang**: Data curation; methodology; visualization. **Hanna K. Mandl**: Data curation; formal analysis; methodology. **Jyothi K. Rajashekar**: Data curation; methodology. **Krasimir A. Spasov**: Data curation; formal analysis; methodology. **William L. Jorgensen**: Conceptualization; funding acquisition; investigation; project administration; resources; supervision; writing‐original draft; writing‐review and editing. **W. Mark Saltzman**: Conceptualization; funding acquisition; project administration; resources; writing‐original draft; writing‐review and editing. **Karen S. Anderson**: Conceptualization; funding acquisition; investigation; project administration; resources; supervision; writing‐original draft; writing‐review and editing. **Priti Kumar**: Conceptualization; funding acquisition; investigation; project administration; resources; supervision; writing‐original draft; writing‐review and editing.
